# Supplementary material for: Unveiling the molecular mechanisms of the type IX secretion system's response regulator: Structural and functional insights
Source: PNAS Nexus. 2024 Jul 31;3(8):pgae316. doi: 10.1093/pnasnexus/pgae316 (PMC11320123; doi:10.1093/pnasnexus/pgae316)
Supplement: pgae316_Supplementary_Data [file pgae316_supplementary_data.pdf]

Supporting Information:

Unveiling the Molecular Mechanisms of the Type-IX Secretion System's Response Regulator: Structural and Functional Insights

Anshu Saran[1,2], Hey-Min Kim[3], Ireland Manning[4], Mark A. Hancock[5,2], Claus Schmitz[6], Mariusz Madej[7], Jan Potempa[7,8], Maria Sola[6], Jean-François Trempe[5,2], Yongtao Zhu[4,9], Mary Ellen Davey[3], Natalie Zeytuni[1,2 †]

[1] Department of Anatomy and Cell Biology, McGill University, Montreal, Quebec, Canada.
Address: 3640 Rue University, Montreal, Quebec, Canada H3A 0C7

[2] Centre de Recherche en Biologie Structurale (CRBS), Montreal, Quebec, Canada.

[3] Department of Microbiology, The Forsyth Institute, Cambridge, MA 02142 USA

[4] Department of Biological Sciences, Minnesota State University Mankato, Mankato, Minnesota, USA

[5] Department of Pharmacology & Therapeutics, McGill University, Montreal, Quebec, Canada, H3G 1Y6.

[6] Department of Structural Biology, Molecular Biology Institute of Barcelona, Spanish Research Council, Barcelona Science Park, Barcelona, E-08028, Spain.

[7] Department of Microbiology, Faculty of Biochemistry, Biophysics and Biotechnology, Jagiellonian University, Krak´ow PL-30-387, Poland.

[8] Department of Oral Immunology and Infectious Diseases, School of Dentistry, University of Louisville, Louisville, Kentucky 40202, USA.

[9] Department of Biological Sciences, Xi'an Jiaotong-Liverpool University, Suzhou, Jiangsu, China.

[†] Email address for correspondence: natalie.zeytuni@mcgill.ca

Table S1 - Primers used in this study.

**Primer**	**Sequence (5' --> 3')**	**Purpose**
porX$_{FJ}$ fw	CAGCGGCCTGGTGCCGCGCGGCAGCCATATGGATAAGATAAGAATACTTTGGGTCGATG	pET28a-*porX*$_{FJ}$
porX$_{FJ}$ rev	TTGTCGACGGAGCTCGAATTCGGATCCTTATTTAGGGTTAAATACCAAAAACGG	pET28a-*porX*$_{FJ}$
pET28 Kan fw	CCCATTTATACCCATATAAATCAGCATCCATGTTGGAATTTAATCGCGGC	*porX*$_{FJ}$ mutant construction by Gibson assembly
pET28 Kan rev	GCCGCGATTAAATTCCAACATGGATGCTGATTTATATGGGTATAAATGGG	*porX*$_{FJ}$ mutant construction by Gibson assembly
porX$_{FJ}$ L113E fw	ACCAGTAAATCCAAATCAAATTTTAGAGAGTTTAAAAAAGAATCTGGATG	pET28a-*porX*$_{FJ}$ L113E
porX$_{FJ}$ L113E rev	CATCCAGATTCTTTTTTAAACTCTCTAAAATTTGATTTGGATTTACTGGT	pET28a-*porX*$_{FJ}$ L113E
porX$_{FJ}$ T271V fw	GTATTTCTCTATTCTTCCAACTGCAGTACAATATGCCAGAAATGCAATTTTC	pET28a-*porX*$_{FJ}$ T271V
porX$_{FJ}$ T271V rev	GAAAATTGCATTTCTGGCATATTGTACTGCAGTTGGAAGAATAGAGAAATAC	pET28a-*porX*$_{FJ}$ T271V
porX$_{FJ}$ D54A fw	GAAGAAAACTTTGACATTGTTTTTCTTGCCGAAAATATGCCGGGAATGAGCG	pET28a-*porX*$_{FJ}$ D54A
porX$_{FJ}$ D54A rev	CGCTCATTCCCGGCATATTTTCGGCAAGAAAAACAATGTCAAAGTTTTCTTC	pET28a-*porX*$_{FJ}$ D54A
porX$_{FJ}$ K104A fw	CTAAAATCGCAGACTATTTGATAGCACCAGTAAATCCAAATCAAATTTTAC	pET28a-*porX*$_{FJ}$ K104A
porX$_{FJ}$ K104A rev	GTAAAATTTGATTTGGATTTACTGGTGCTATCAAATAGTCTGCGATTTTAG	pET28a-*porX*$_{FJ}$ K104A
porX$_{FJ}$ D360A/H364A fw	GTGACTGTTGTTTATAATTTCGTTGCTATGCTTTCGGCTGCAAAAACTGAAATGGAAGTT	pET28a-*porX*$_{FJ}$ D360A/H364A
porX$_{FJ}$ D360A/H364A rev	AACTTCCATTTCAGTTTTTGCAGCCGAAAGCATAGCAACGAAATTATAAACAACAGTCAC	pET28a-*porX*$_{FJ}$ D360A/H364A
porX$_{FJ}$ S384A/S388E rev	ATAATGGAGAATTTTTAAACCATTCTAAAGTCAGTGCGCGATATGCTTTGTCATCAGAAG	pET28a-*porX*$_{FJ}$ S384A/S388E
porX$_{FJ}$ S384A/S388E fw	CTTCTGATGACAAAGCATATCGCGCACTGACTTTAGAATGGTTTAAAAATTCTCCATTAT	pET28a-*porX*$_{FJ}$ S384A/S388E

porX$_{FJ}$ stop codon after 121 fw	ACTGAGTTTAAAAAAGAATCTGGATGATTAATCAAGACTGATTACAGAAAAAAC	pET28a-*porX*$_{FJ}$ REC
porX$_{FJ}$ stop codon after 121 rev	GTTTTTTCTGTAATCAGTCTTGATTAATCATCCAGATTCTTTTTTAAACTCAGT	pET28a-*porX*$_{FJ}$ REC
porX$_{FJ}$ stop codon after 208 fw	CTGGTTTGCTCCAAAAGCAGATAAATAACCAATTCAATCTCATAATTTATTTAAAG	pET28a-*porX*$_{FJ}$ REC+THB
porX$_{FJ}$ stop codon after 208 rev	CTTTAAATAAATTATGAGATTGAATTGGTTATTTATCTGCTTTTGGAGCAAACCAG	pET28a-*porX*$_{FJ}$ REC+THB
porX$_{FJ}$ 126-end fw	CCGCGCGGCAGCCATATGACAGAAAAAACAACATTAGATTACCAAAAAGAATTC	pET28a-*porX*$_{FJ}$ THB+APS
porX$_{FJ}$ 126-end rev	CTGGTTTGCTCCAAAAGCAGATAAATAACCAATTCAATCTCATAATTTATTTAAAG	pET28a-*porX*$_{FJ}$ THB+APS
FJ0076	GCTAGGGATCCAACATTATCCCCCAAAACG	Δ*porX*$_{FJ}$
FJ0077	GCTAGTCTAGAATTGTTGCTTGTTGTAACTTC	Δ*porX*$_{FJ}$
FJ0167	GCTAGTCTAGATTAGAAGAAATGATTATTCCGTTTT	Δ*porX*$_{FJ}$
FJ0079	GCTAGGTCGACGATTTTACAGCTGGATAAGAAC	Δ*porX*$_{FJ}$
FJ0086	GCTAGGGATCCGATGAAAACGTGTATGATTTG	Identification of Δ*porX*$_{FJ}$ CJ4057
FJ0087	GCTAGTCTAGACAGTCACAGTTTTCACTTCT	Identification of Δ*porX*$_{FJ}$ CJ4057
FJ0088	GCTAGGGATCCGATGAAAACGTGTATGATTTG	pIM10-*porX*
FJ0089	GCTAGTCTAGACAGTCACAGTTTTCACTTCT	pIM10-*porX*
FJ0734	AATGATGTCGACTCTGCGCTTCCTATTTCG	Δ*gldKLMNO*
FJ0735	CATATCGCATGCTTTCATACGATTTGTATCTGTAGCTGC	Δ*gldKLMNO*
FJ1209	GCTAGGGATCCGCCAATTGCTGTTTACAAAGGAG	Δ*gldKLMNO*
FJ1210	GCTAGGTCGACACCTGACTTACCACAGCCGAT	Δ*gldKLMNO*
W50 Δ*porX*$_{PG}$ F1 fw	TTGTAAAACGACGGCCAGTGAATTCTTGGCGACACGCGGACTC	Δ*porX*$_{PG}$::Erm
W50 Δ*porX*$_{PG}$ F1 rev	TCTTTTTTGTCATAATTATGTTCTTCTCTATTTAGTATAGGGTATAACGGAAGTG	Δ*porX*$_{PG}$::Erm
W50 Δ*porX*$_{PG}$ F2 fw	AAGAACATAATTATGACAAAAAAGAAATTGCC	Δ*porX*$_{PG}$::Erm

W50 Δ*porX*$_{PG}$ F2 rev	TGTATGAAGTATCTACGAAGGATGAAATTTTTC	Δ*porX*$_{PG}$::Erm
W50 Δ*porX*$_{PG}$ F3 fw	TCATCCTTCGTAGATACTTCATACATGAATACGATC	Δ*porX*$_{PG}$::Erm
W50 Δ*porX*$_{PG}$ F3 rev	CTATGACCATGATTACGCCAAGCTTCAGTATATTGGCC GAATTG	Δ*porX*$_{PG}$::Erm
pUC19 sequencing fw	TACGCCAGCTGGCGAAAGGGGGATG	Δ*porX*$_{PG}$::Erm sequencing
pUC19 sequencing rev	GCTTTACACTTTATGCTTCCGGCTCG	Δ*porX*$_{PG}$::Erm sequencing
W50 Δ*porX*$_{PG}$ fw	ATCACAACGCGAACACCCTGATC	Δ*porX*$_{PG}$::Erm sequencing
W50 Δ*porX*$_{PG}$ rev	CCACAGAGGATATATTCGGATAG	Δ*porX*$_{PG}$::Erm sequencing
pTMCS groES fw	CGATAAGCTTGGATCCGCATGCCCCATTGGATAGATGC CCTGC	pTCOW-groES-*porX*$_{PG}$
groES PorX$_{PG}$ rev	GTTTTTTTCCATTGTTGCTTGGTTTGTTATTG	pTCOW-groES-*porX*$_{PG}$
groES *porX*$_{PG}$ fw	AAACCAAGCAACAATGGAAAAAAACATGAGACC	pTCOW-groES-*porX*$_{PG}$
porX$_{PG}$ pTMCS rev	TAGCGAGGTGCGGCCGGTCGACCCCTTACTTGGGTTGC ATCGTAATTAC	pTCOW-groES-*porX*$_{PG}$
groES *porX*$_{FJ}$ F1 rev	TTATCTTATCCATTGTTGCTTGGTTTGTTATTG	pTCOW-groES-*porX*$_{FJ}$
groES *porX*$_{FJ}$ F2 fw	AACCAAGCAACAATGGATAAGATAAGAATACTTTGG	pTCOW-groES-*porX*$_{FJ}$
porX$_{FJ}$ F2 RV	TAGCGAGGTGCGGCCGGTCGACCCCTTATTTAGGGTTA AATACCAAAAAC	pTCOW-groES-*porX*$_{FJ}$

$porX_{PG}$ D58A rev	GTCCGCCGATGCCGGGCATGTTCTCAGCGAGGAATAC GATGTCGAAGTCG	pTCOW-groES-$porX_{PG}$ D58A
$porX_{PG}$ D58A fw	CGACTTCGACATCGTATTCCTCGCTGAGAACATGCCCG GCATCGGCGGAC	pTCOW-groES-$porX_{PG}$ D58A
$porX_{PG}$ T272V rev	AGATGGCATTGCGTGCATATTGGACCGCTGTCGGCAGG ATGGACAGGTAC	pTCOW-groES-$porX_{PG}$ T272V
$porX_{PG}$ T272V fw	GTACCTGTCCATCCTGCCGACAGCGGTCCAATATGCAC GCAATGCCATCT	pTCOW-groES-$porX_{PG}$ T272V
$porX_{PG}$ D361A/H365A fw	GATAGTCCTGAACTTCGTGGCCATGATGTCGGCTGCTC GTACTGATAGCAAGATGATTC	pTCOW-groES-$porX_{PG}$ D361A/H365A
$porX_{PG}$ D361A/H365A rev	GAATCATCTTGCTATCAGTACGAGCAGCCGACATCATG GCCACGAAGTTCAGGACTATC	pTCOW-groES-$porX_{PG}$ D361A/H365A
$porX_{PG}$ S385A/S389E fw	GGCATCCAACGAAGCAGCCTATCGCGCGCTGACGAAG GAATGGTTCAAGCATTCGAC	pTCOW-groES-$porX_{PG}$ S385A/S389E
$porX_{PG}$ S385A/S389E rev	GTCGAATGCTTGAACCATTCCTTCGTCAGCGCGCGATA GGCTGCTTCGTTGGATGCC	pTCOW-groES-$porX_{PG}$ S385A/S389E
$porX_{PG}$ L117E rev	TTTTTTGAGCGACTCGAGGAGCTGATTCGGATTC	pTCOW-groES-$porX_{PG}$ L117E
$porX_{PG}$ L117E fw	TCAGCTCCTCGAGTCGCTCAAAAAAAACCTG	pTCOW-groES-$porX_{PG}$ L117E
$porX_{PG}$ stop codon after 125	TAGCGAGGTGCGGCCGGTCGACCCCTTACTGCTGCAGG TTTTTTTTG	pTCOW-groES-$porX_{PG}$ REC
$porX_{PG}$ stop codon after 207	TAGCGAGGTGCGGCCGGTCGACCCCTTACTTGGCAATC CATTCCCGATAG	pTCOW-groES-$porX_{PG}$ REC+THB
$porX_{PG}$ 130-end fw	AACAATGAGCGAAACCACGAACACGAACTACCGGCAA GAGTTCGTCCAAC	pTCOW-groES-$porX_{PG}$ THB+APS
$porX_{PG}$ 130-end rev	CGTGTTCGTGGTTTCGCTCATTGTTGCTTGGTTTGTTAT TGTTAGTTGATTGTTTG	pTCOW-groES-$porX_{PG}$ THB+APS
pTCOW Amp fw	CAGCATCTTTTACTTTCACCAGCGTTTCTGGGTGAGCA AAAACAGGAAGGC	$porX_{PG}$ mutant construction by Gibson assembly

pTCOW Amp rev	GCCTTCCTGTTTTTGCTCACCCAGAAACGCTGGTGAAAGTAAAAGATGCTG	*porX*$_{PG}$ mutant construction by Gibson assembly
pTCOW seq FW	CAGTGAGGATATTGACGCTTATTTCG	groES-porX sequencing
pTCOW seq RV	CGCATTCACAGTTCTCCGCAAG	groES-porX sequencing

Table S2 - Strains and plasmids used in this study.

**Strain (relevant genotype)**	**Source or reference**
***E. coli* strains**	
NEB5α	NEB
DH5αMCR	Life Technologies
BL21	Life Technologies
S17-1	(1)
HB101	(2, 3)
F. johnsoniae strains	
UW101	(4, 5)
UW101 *Δ*$porX_{FJ}$	This study
UW101 *ΔgldKLMNO*	This study
***P. gingivalis* strains**	
W50 (wild type)	ATCC
Δ$porX_{PG}$:Erm (Em^r) in strain W50	This study
ΔporN in strain W83	(6)
**Plasmids**	
pET28a (Kan^R)	Novagen
pRK2013 (IncP Tra$^+$ Km^r)	(3)
pYT313 (Ap^r (Em)^r)	(7)
pYT377 (1.9 kb region upstream of *gldK* cloned in pYT313)	This study
pYT379 (3 kb region downstream of *gldO* cloned in pYT377)	This study
pIM03 (2 kb region upstream of $porX_{FJ}$ cloned in pYT313)	This study
pIM06 (1.9 kb region downstream of $porX_{FJ}$ cloned in pIM03)	This study
pCP23 (Ap^r (Tc)^r)	(8)
pIM10 (2.0 kb region spanning $porX_{FJ}$ cloned in pCP23)	This study
pUC19 (Amp^R)	NEB
pT-COW (Amp^R and Tc^R in *E. coli*; Tc^R in *P. gingivalis*; Mob$^+$ Rep$^+$)	(1)
pVA2198 (Em^r and Sp^r)	(2)
pTCOW-groES-$porX_{PG}$	This study
pTCOW-groES-$porX_{FJ}$	This study
pTCOW-groES-$porX_{PG}$ D58A	This study
pTCOW-groES-$porX_{PG}$ T272V	This study
pTCOW-groES-$porX_{PG}$ D58A/T272V	This study
pTCOW-groES-$porX_{PG}$ D361A/H365A	This study

pTCOW-groES-*porX*$_{PG}$ S385A/S389E	This study
pTCOW-groES-*porX*$_{PG}$ L117E	This study
pTCOW-groES-*porX*$_{PG}$ REC (1-125)	This study
pTCOW-groES-*porX*$_{PG}$ REC+THB (1-207)	This study
pTCOW-groES-*porX*$_{PG}$ THB+APS (130-end)	This study
pET28a-*porX*$_{FJ}$	This study
pET28a-*porX*$_{FJ}$ D54A	This study
pET28a-*porX*$_{FJ}$ T271V	This study
pET28a-*porX*$_{FJ}$ D54A/T271V	This study
pET28a-*porX*$_{FJ}$ D360A/H364A	This study
pET28a-*porX*$_{FJ}$ S384A/S388E	This study
pET28a-*porX*$_{FJ}$ L113E	This study
pET28a-*porX*$_{FJ}$ K104A	This study
pET28a-*porX*$_{FJ}$ REC (1-121)	This study
pET28a-*porX*$_{FJ}$ REC+THB (1-208)	This study
pET28a-*porX*$_{FJ}$ THB+APS (126-end)	This study

Table S3 - Crystallization conditions and data collection parameters

	**PorX$_{FJ}$ -SO$_4$**	**PorX$_{FJ}$-Br (also used for phasing)**	**PorX$_{FJ}$-BeF$_3$**	**PorX$_{FJ}$-Zn**	**PorX$_{FJ}$-T271V**
PDB code	8TEF	8TED	8TFF	8TFM	8THP
Crystallization condition	0.2 M Lithium sulfate, 0.1 M Tris pH 8.1 and 35% polyethylene glycol 400	0.16 M calcium acetate, 0.08 M sodium cacodylate pH 6.5, 14.4% polyethylene glycol 8000 and 20% glycerol (0.5 M NaBr in the drop)	0.16 M calcium acetate, 0.08 M sodium cacodylate pH 6.5, 14.4% polyethylene glycol 8000 and 20% glycerol (supplemented with 166 μM beryllium sulphate, 1.162 mM Sodium fluoride and 83 μM Manganese chloride in the drop)	0.16 M calcium acetate, 0.08 M sodium cacodylate pH 6.5, 14.4% polyethylene glycol 8000, 25% glycerol and 0.2 mM Zinc chloride	0.16 M calcium acetate, 0.08 M sodium cacodylate pH 6.14, 14.4% polyethylene glycol 8K, 20% Glycerol

Beamline	ALS502, Lawrence Berkeley National Laboratory Advanced Light Source (ALS)	CMCF-BM, Canadian Light Source (CLS)	ALS501, Lawrence Berkeley National Laborator y Advanced Light Source (ALS)	CMCF-ID, Canadian Light Source (CLS)	CMCF-ID, Canadian Light Source (CLS)
Detector	Pilatus3 6M 25 Hz	Pilatus3 6M 25 Hz	Pilatus3 2M 25 Hz	Eiger X 9M	Eiger X 9M
Detector distance (mm)	400	347	400	152.3	223.2
Wavelength (Å)	0.9823	0.919 (peak)<br>0.953 (remote)	0.9774	1.283	0.953
Oscillation range (°)	0.25	0.5 (peak)<br>0.5 (remote)	0.25	0.2	0.2
Time of exposure per image (s)	0.25	2 (peak)<br>1 (remote)	0.5	0.02	0.02
Number of images	1440	800 (peak)<br>360 (remote)	720	1800	900

Table S4 - Data refinement statistics of the crystals. Statistic values present in parentheses correspond to the highest resolution shell data.

	**PorX$_{FJ}$-SO$_4$**	**PorX$_{FJ}$-Br (also used for phasing)**	**PorX$_{FJ}$-BeF$_3$**	**PorX$_{FJ}$-Zn**	**PorX$_{FJ}$-T271V**
PDB code	8TEF	8TED	8TFF	8TFM	8THP
Space group	$P2_1$	$P2_12_12_1$	$P2_12_12_1$	$P2_12_12_1$	$P2_12_12_1$
Number of molecules per asymmetric unit	4	2	2	2	2
**Cell dimensions**					
a, b, c (Å)	133.52, 57.52, 149.14	84.70, 97.85, 133.20	84.32, 97.52, 131.26	86.87, 97.06, 132.04	86.09, 97.98, 130.94
α, β, γ (°)	90.00, 98.39, 90.00	90.00, 90.00, 90.00	90.00, 90.00, 90.00	90.00, 90.00, 90.00	90.00, 90.00, 90.00
Resolution (Å)	46.00-2.85 (2.90-2.85)	46.16-2.10 (2.15-2.10)	50.00-2.34 (2.38-2.34)	50-2.72 (2.77-2.72)	50-2.6(2.64-2.6)
Rsym or Rmerge (%)	10.30 (32.50)	8.40 (52.70)	10.80 (42.20)	11.3 (82.9)	9.6 (36.7)
R meas (%)	11.90 (37.90)	9.90 (62.10)	11.80 (46.00)	11.8 (87.0)	10.8 (40.9)
Rpim (%)	5.80 (19.10)	5.20 (32.60)	4.60 (18.20)	3.3 (25.7)	4.70(17.9)
I / σI	12.20 (3.85)	13.70 (3.50)	17.31 (3.25)	24.11(2.22)	18.72 (3.19)
Completeness (%)	100 (100)	99.60 (99.10)	100(100)	99.8(97.7)	99.9 (99.8)
Redundancy	4	6.7	6.6	12.7	5
CC1/2	0.984 (0.891)	0.998 (0.920)	0.980 (0.910)	0.998 (0.901)	0.990 (0.865)
CC*	0.996 (0.971)	0.999 (0.978)	0.996 (0.978)	0.999 (0.974)	0.998 (0.963)
Wavelength (Å)	0.982	0.953	0.977	1.283	0.953
**Refinement**					
Resolution (Å)	45.40-2.85	42.40-2.10	48.81-2.34	48.58-2.72	45.95-2.60
No. reflections (unique)	53000	65014	46485	31924	34201
Rwork / Rfree (%)	17.67/22.16	17.86/22.56	16.51/23.10	20.43/26.11	24.97/30.46
**No. of atoms**					
Protein	16797	8481	8476	8282	8271
Ligand/ion	59	33	27	18	10
Water	56	686	381	26	44

**B-factors**					
Protein	51.269	33.187	38.540	43.062	52.464
Ligand/ion	66.654	49.76	48.698	82.374	41.502
Water	27.765	37.107	35.647	50.907	25.928
MolProbity score	1.40	1.32	1.39	1.84	1.65
Rotamer outliers (%)	1.92	1.37	1.69	3.04	2.07
Ramachandran favoured (%)	97.78	97.53	97.35	96.98	98.08
Ramachandran allowed (%)	2.22	2.47	2.65	2.92	1.92
Ramachandran outliers	0	0	0	0.1	0
**R.m.s. deviations**					
Bond lengths (Å)	0.0056	0.0084	0.0075	0.0062	0.0057
Bond angles (°)	1.238	1.484	1.378	1.329	1.067

<u>Table S5</u> – Intact protein LC-MS analyses of PorX$_{FJ}$ variants in the absence or presence of phosphorylation *in vitro*. Expected molecular weights for protein variants (theoretical averages) vs. observed peaks before or after AcP/Mg^{2+} reaction (+ 79.9 single phosphorylation; + 159.8 dual phosphorylation; - 18.0 dehydration; + 61.9 combined dehydration with single phosphorylation).

	Non-phosphorylated (-AcP)		Phosphorylated (+AcP)							
			Single phosphorylation		Dual phosphorylation		Cyclization (dehydration)		Cyclization (dehydration) + single phosphorylation	
	Expected	Observed	Expected (+79.9)	Observed	Expected (+159.8)	Observed	Expected (-18.0)	Observed	Expected (-18.0+79.9)	Observed
**WT**	60449.9	60449.1	60529.8	60528.4 (+79.3)	60609.7	60609.7 (+160.6)	60431.9	60430.9 (-18.2)	60511.8	60511.5
**D54A**	60405.9	60405.2	60485.8	60485.5 (+80.3)	–	–	–	–	–	–
**T271V**	60447.9	60447.4	60527.8	60527.8 (+80.4)	–	–	60429.9	60429.3 (-18.1)	–	–
**D54A/ T271V**	60403.9	60403.5	–	–	–	–	–	–	–	–
**D360A/ H364A**	60339.8	60338.9	60419.7	60419.4 (+80.5)	–	–	60321.8	60321.2 (-17.7)	–	–
**L113E**	60465.8	60465.1 60545.4 (at T271)*	60545.7	60545.3 (+80.2)	60625.6	60626.3 (+161.2)	60447.8	60447.2 (-17.9)	60527.7	60527.0
**S384A/ S388E**	60475.9	60475.9	60555.8	60555.5 (+79.6)	60635.7	60636.7 (+160.8)	60457.9	60457.7 (-18.2)	60537.8	60537.4
**K104A**	60392.8	60392.2	60472.2	60472.8 (+80.6)	–	–	–	–	–	–
**REC**	14203.4	14202.8	14283.3	14282.7 (+79.9)	–	–	14185.4	14185.1 (-17.7)	–	–
**REC+THB**	24762.4	24761.7	24842.3	24841.9 (+80.2)	–	–	24744.4	24743.6 (-18.1)	–	–
**THB+APS**	46208.0	46207.0	46287.9	46286.9 (+79.9)	–	–	–	–	–	–

*Note: Peak also observed at 60545.4 for L113E variant was likely due to pre-existing phosphorylation at T271 prior to incubation with acetyl phosphate (AcP).

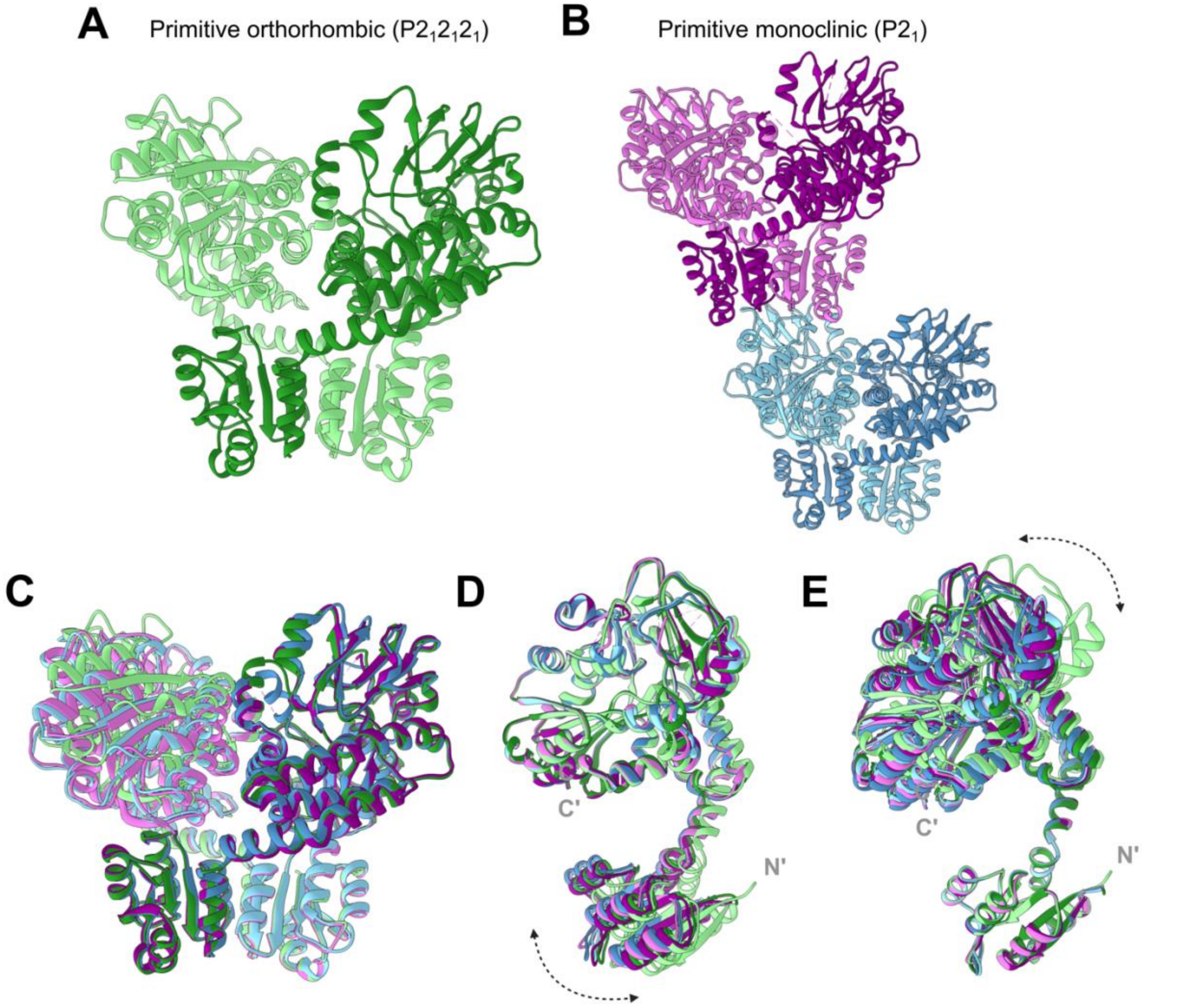

<u>Fig. S1</u> - PorX$_{FJ}$ structure in two crystal forms. Asymmetric unit composition of **(A)** primitive orthorhombic and **(B)** primitive monoclinic crystal forms. **(C)** Superposition of the three dimers shown in panels A and B. Overlay of the six monomers shown in A and B aligned according to their **(D)** alkaline phosphatase superfamily (APS) domain and **(E)** receiver (REC) domain.

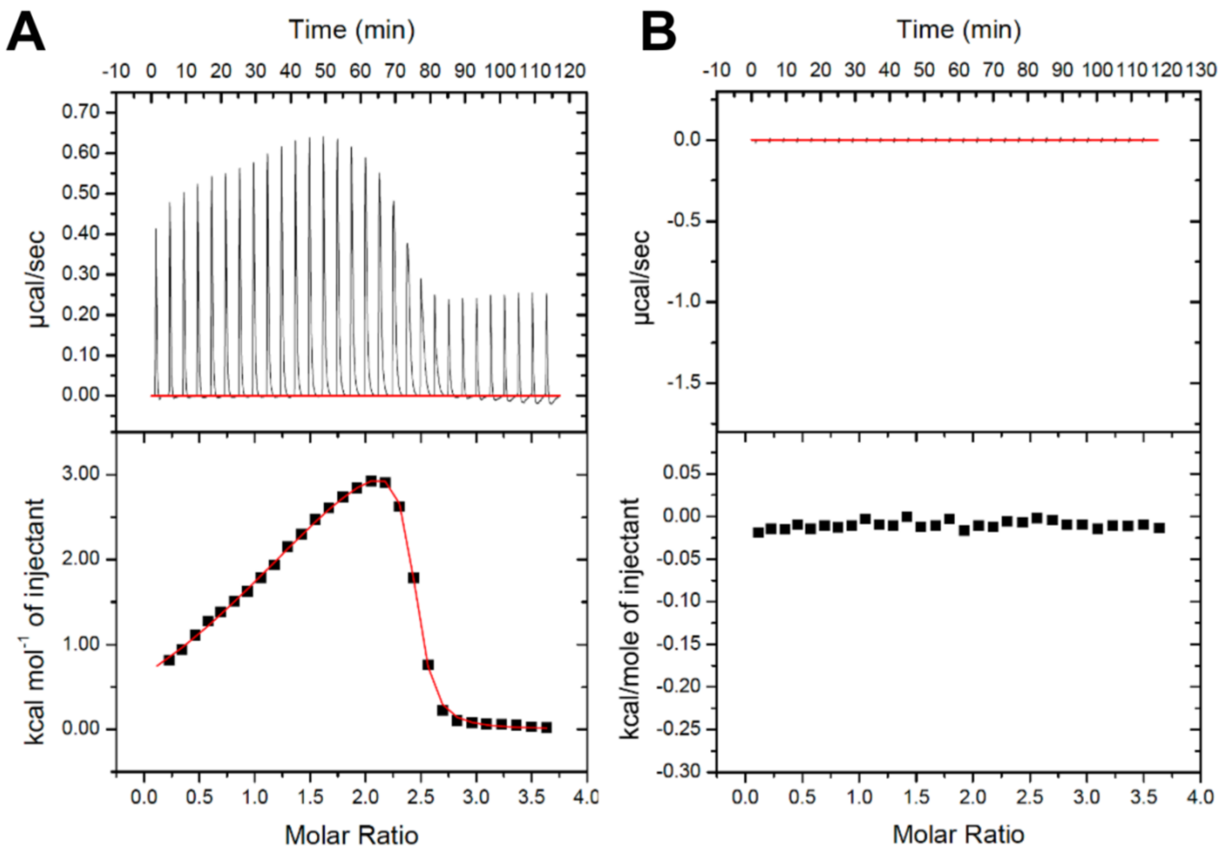

Fig. S2 - **Zinc binding to PorX$_{FJ}$ by isothermal titration calorimetry. (A)** Binding isotherm of zinc titrated into PorX$_{FJ}$ reveals two zinc sites stoichometry with N_1= 1.09 ± 0.04 sites, K_{D1}=180.83 ± 14.93 nM, ΔH_1=-2.374E4 ± 1.28E6 cal/mol, ΔS_1=-50.2 cal/mol/deg and N_2=1.19 ± 0.04 sites, K_{D2}= 59.17 ± 13.81 nM, ΔH_2=2.744E4 ± 1.26E6 cal/mol, ΔS_2=124 cal/mol/deg. **(B)** Buffer titrated into PorX$_{FJ}$ as a control.

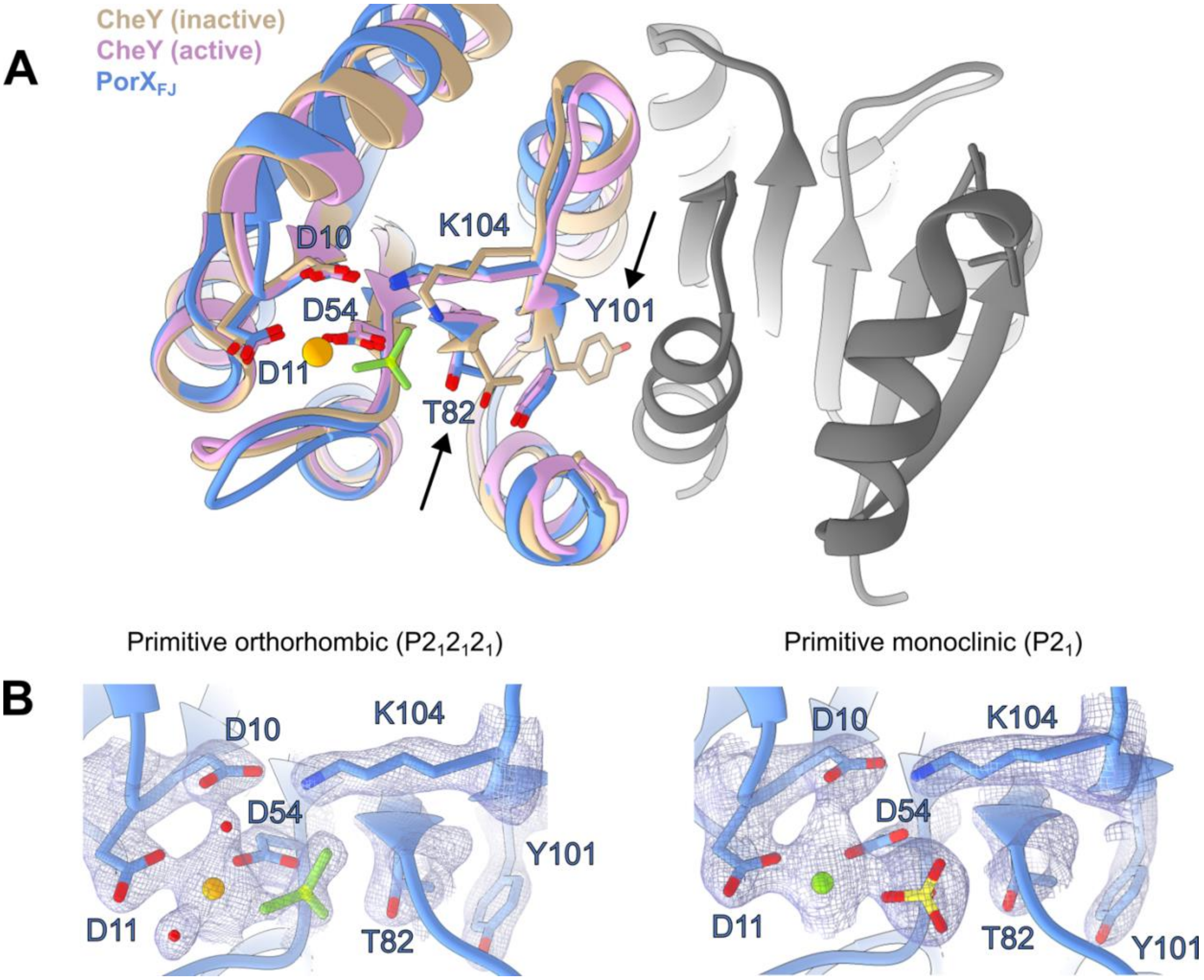

Fig. S3 – The crystal structure of PorX$_{FJ}$ adopts the active phosphorylated-like conformation. **(A)** Superposition of the primitive orthorhombic crystal form of PorX$_{FJ}$ with the active (PDB ID:1FQW) and inactive (PDB ID: 2CHE) conformations of the CheY response regulator reveal an active-like conformation of PorX$_{FJ}$, as per the positioning of the conserved Thr-Tyr pair, marked by arrows. Inactive and active conformations of CheY are colored in tan and pink, while chains A and B of PorX$_{FJ}$ dimer are colored in blue and grey, respectively. Specific residue labels are according to PorX$_{FJ}$ sequence. **(B)** Electron density of phosphate analogs, BeF_3 and SO_4^{2-}, at the REC domain phosphorylation site in the different crystal forms of PorX$_{FJ}$. Orange, green and red spheres represent Ca^{2+}, Mg^{2+} and H_2O respectively.

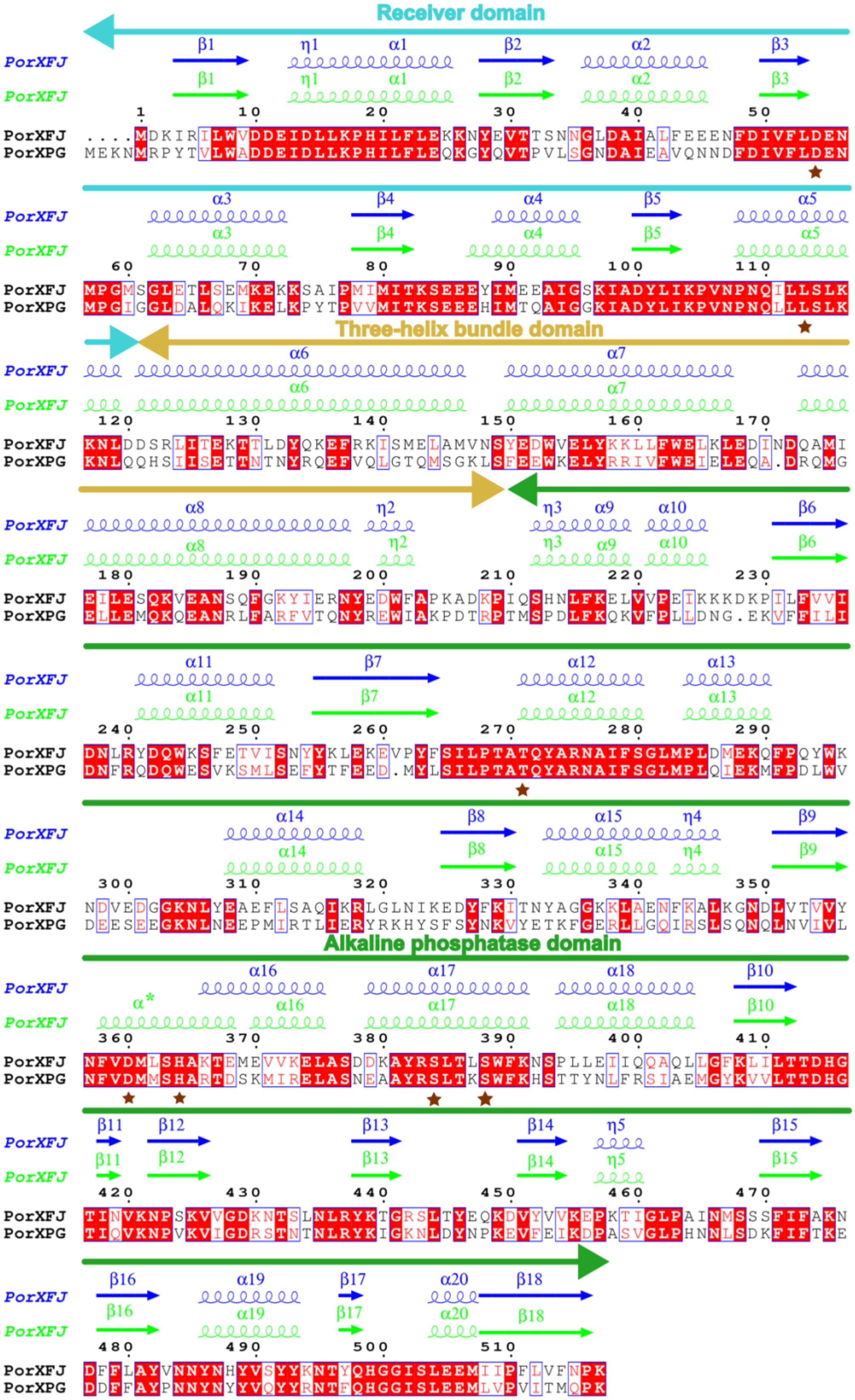
Receiver domain
Three-helix bundle domain
Alkaline phosphatase domain
PorXFJ
PorXPG

<u>Fig. S4</u> - Multiple sequence alignment of PorX$_{FJ}$ and PorX$_{PG}$. The alignment was prepared using Clustal Omega (9) and ESPript (10). The receiver, three-helix bundle and alkaline phosphatase domains are labelled in cyan, yellow and green respectively. Reference amino acid numbering and secondary structure prediction is according to the PorX$_{FJ}$ sequence. The secondary structure alignment in blue and light green correspond to the alternate chain conformations of the primitive orthorhombic dimeric structure with α* indicating the area of conformational difference between the two chains. Amino acid residues marked with a star correspond to functionally significant residues whose mutations are discussed in this manuscript.

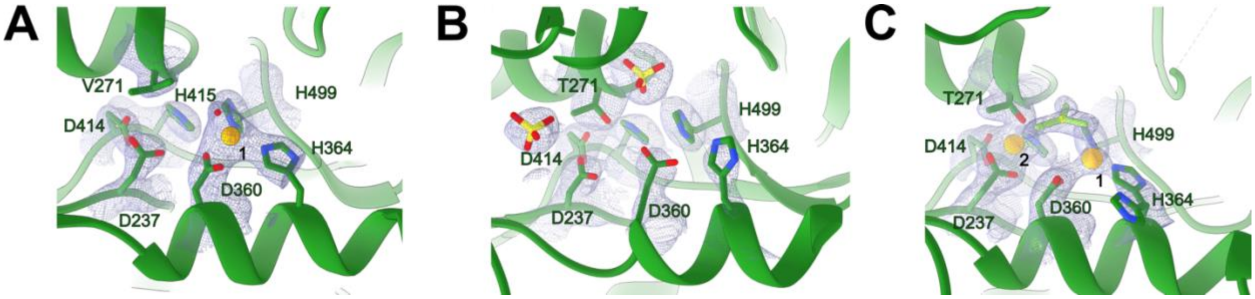

<u>Fig. S5</u> – Electron density of divalent cations and phosphate analogs in the APS domain of **(A)** PorX$_{FJ}$-T271V crystal form **(B)** PorX$_{FJ}$-SO_4^{2-} primitive monoclinic crystal form and **(C)** PorX$_{FJ}$-BeF_3 primitive orthorhombic crystal form**.**

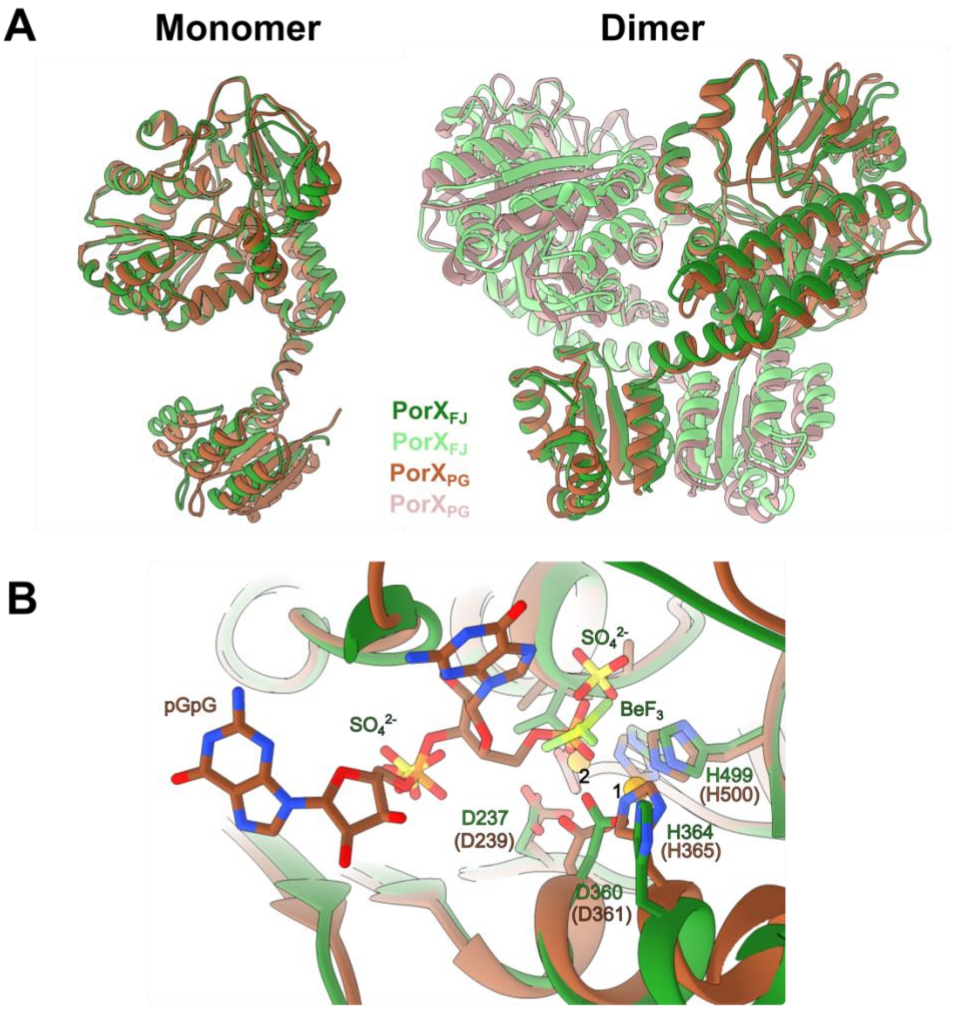

Fig. S6 – The structures of PorX$_{FJ}$ and PorX$_{PG}$ (PDB code:7PVK) exhibit a highly conserved fold. (A) A superimposition of a representative monomer and a representative dimeric assembly. **(B)** Superimposition of the active site of the APS domain. Ligand analogs BeF_3 and sulphate ions observed in the PorX$_{FJ}$ structures directly overlap or are situated in close proximity to the phosphoguanylyl-(3′→5′)-guanosine (pGpG) bound to PorX$_{PG}$.

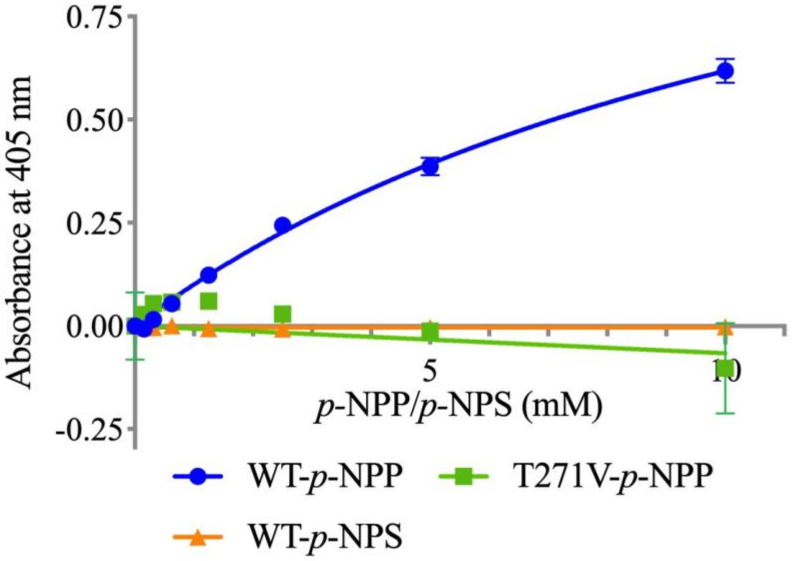

<u>Fig. S7</u> - Monophosphatase and sulphatase activities of PorX$_{FJ}$. Catalytic activities against *p*-nitrophenyl phosphate (*p*-NPP) and *p*-nitrophenyl sulphate (*p*-NPS) substates were recorded in the presence of zinc after three days.

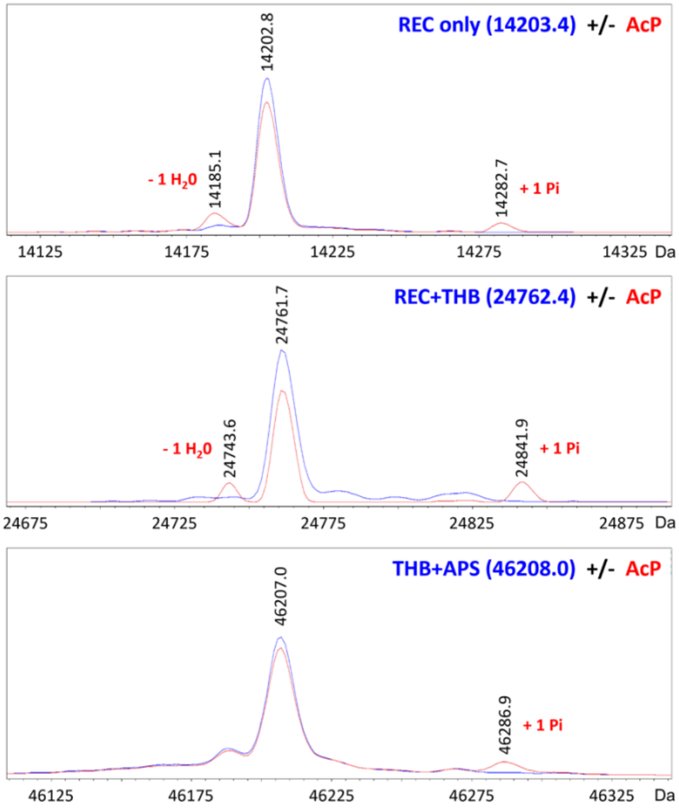

<u>Fig. S8</u> - Intact protein LC-MS analyses of PorX$_{FJ}$ truncation variants in the absence or presence of phosphorylation *in vitro*. The blue and red spectra correspond to non-phosphorylated and phosphorylated versions of the protein, respectively. The acetyl phosphate (AcP) phosphodonor promotes the phosphorylation of Asp54 and/or Thr271 ("+1 Pi" label). Alternatively, AcP was found to induce cyclization of Asp54 and Lys104, resulting in a dehydration reaction ("-1 $H_2$0" labels).

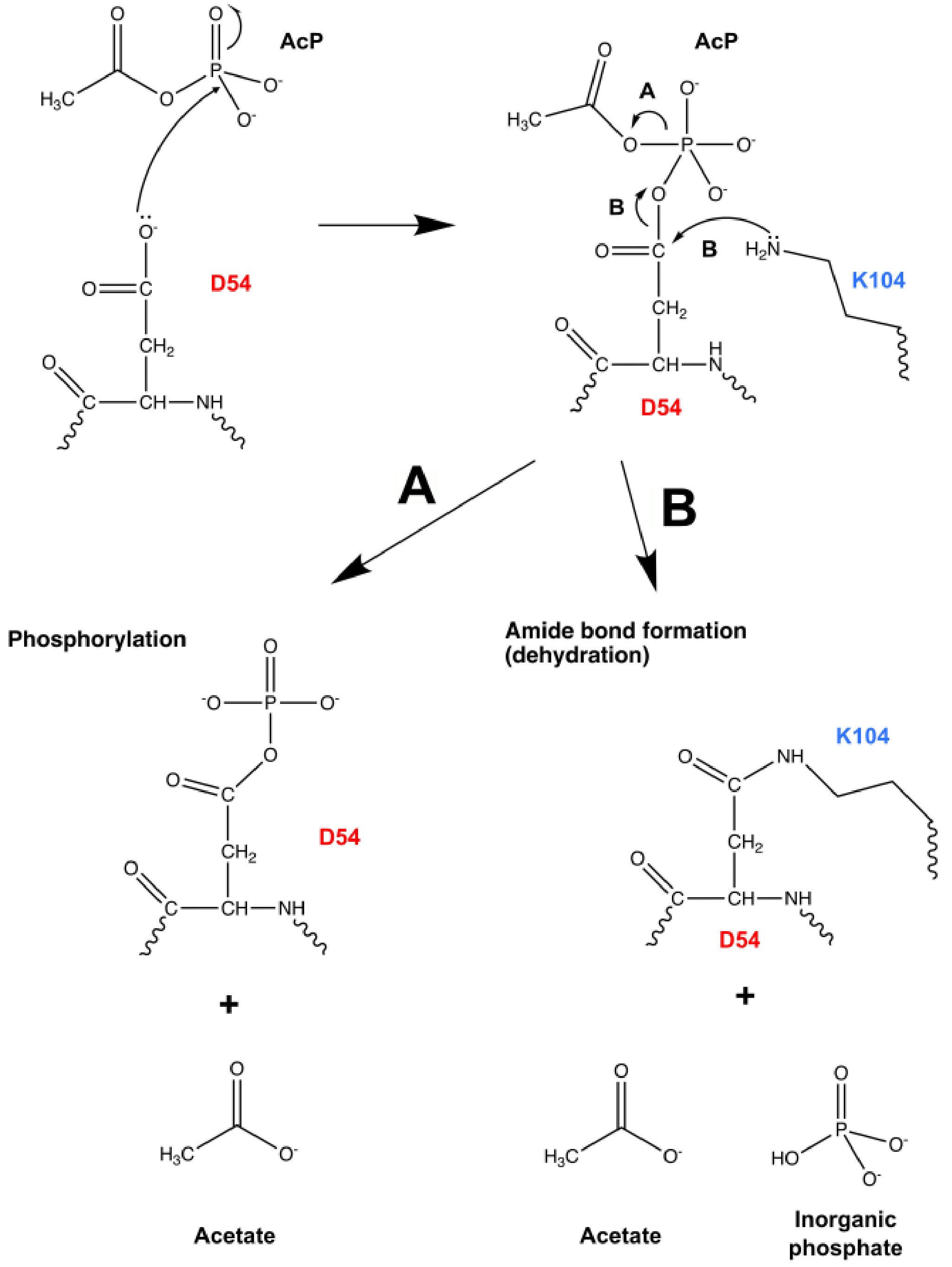

<u>Fig. S9</u> - Proposed phosphorylation mechanism by acetyl phosphate *in vitro*. Reaction of active D54 with AcP can lead to **(A)** phosphorylation and/or **(B)** cyclization involving the formation of an internal peptide linkage between D54 and K104.

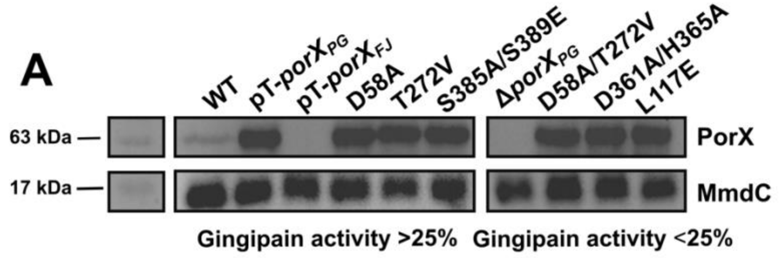

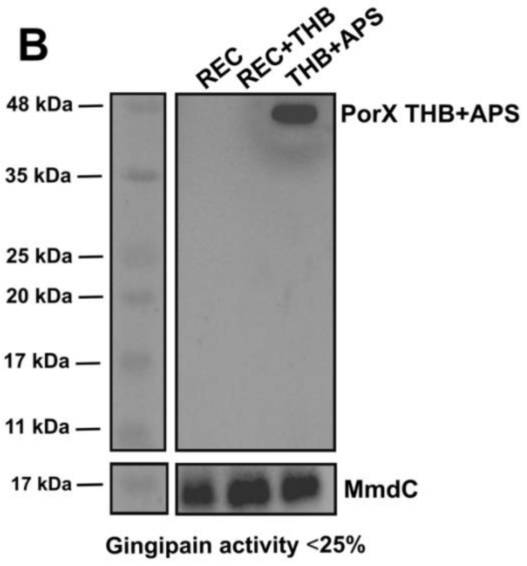

<u>Fig. S10</u> - Expression levels of PorX in *P. gingivalis*. (A) point mutations and (B) truncation variants. Whole cell lysates underwent SDS-PAGE separation followed by Western blot analysis. A rabbit polyclonal anti-PorXPG primary antibody and a polyclonal goat anti-rabbit horseradish peroxidase-conjugated secondary antibody were employed to detect PorX (~61 kDa). The biotinylated protein MmdC (~15 kDa) was used as a loading control (6) and was detected using horseradish peroxidase conjugated Streptavidin.

The anti-PorX$_{PG}$ did not recognize PorX$_{FJ}$ due to sequence variations that affect epitope recognition. Among the truncation variants, PorXPG-REC (~14 kDa) and PorX$_{PG}$-REC+THB (~24 kDa) were not detected, while the PorX$_{PG}$-THB+APS variant (~46 kDa) was identified by the anti-PorX$_{PG}$. This selective recognition of the APS domain truncation variant by the anti-PorX$_{PG}$ antibody may be attributed to the presence of recognizable epitopes exclusively within the APS domain. Nonetheless, the possibility of low expression levels or the instability and subsequent degradation of the PorX$_{PG}$-REC and PorX$_{PG}$-REC+THB truncation variants cannot be excluded.

Detailed methods:

Isothermal titration calorimetry (ITC)

Binding affinity measurements were performed at 20°C using an isothermal titration calorimeter (Microcal iTC200, Malvern). 50 μM $PorX_{FJ}$ was diluted in buffer E and placed in the sample cell. Buffer E supplemented with 750 μM zinc chloride was placed in the syringe and titrated into the protein samples. Each titration was 10 μl in volume and lasted for 10.3 sec followed by an equilibration period of 240 s.

Inductively coupled plasma mass spectrometry (ICP-MS)

$PorX_{FJ}$ variants and method blanks, were digested in PTFE vessels using trace metal grade concentrated nitric acid at room temperature overnight, followed by 2 h at 90°C. Digestates were diluted with deionized water to 2 % w/w nitric acid and the concentrations of Mg, Mn, Co, Zn, and Cd were determined by ICP-MS. A Thermo X- Series 2 ICP-MS with collision cell technology (CCT) and chilled spray chamber was used in kinetic energy discrimination (KED) mode with 8 % hydrogen in helium to reduce interferences. The protein-divalent metal cation stoichiometries were determined by dividing the total number of the protein and each divalent metal cation number of moles.

Phosphodiesterase activity assay *in vitro*

$PorX_{FJ}$ variants (2.5 μM wild-type or mutants) in buffer G (50 mM Tris pH 8, 150 mM sodium chloride) were screened for their phosphodiesterase activity. Similarly, the phosphorylated variants were incubated in buffer G supplemented with 20 mM AcP and 5 mM $MgCl_2$ for 1 h at 37°C prior to measurements. For metal screening, wild-type $PorX_{FJ}$ was incubated in buffer G alone or supplemented with 0.5 mM $ZnCl_2$, $CuCl_2$, $MnCl_2$, $MgCl_2$, or $CaCl_2$. For activity pH screening, phosphorylated and non-phosphorylated $PorX_{FJ}$ were incubated in 150 mM NaCl, 3 μM $ZnCl_2$, supplemented with 50 mM acetic acid (pH 6), 50 mM Tris (pH 7-9) or CAPS buffer (10-11) for 30 min at 37°C. The catalysis of bis(4-nitrophenyl) phosphate (bis-*p*NPP), *p*-nitrophenyl phosphate (*p*NPP) or *p*-nitrophenyl sulphate (*p*NPS) (0-10 mM) and the formation of *p*-nitrophenol product were monitored at 405 nm on a Synergy H1 microplate reader (BioTek). The bis-*p*NPP reaction was monitored for 2 h at 37°C, while the *p*NPP and *p*NPS reactions were monitored for 3 days at 37°C. All assays were performed in quadruplicates. However, the

absorbance measurements of the T271V mutant could not be obtained for bis-*p*NPP concentrations exceeding 5 mM.

Protein phosphorylation assay *in vitro*

For intact protein mass spectrometry, purified $PorX_{FJ}$ variants were incubated at 100 μM in buffer F (25 mM Tris pH 7.5, 10 mM $MgCl_2$, 1 mM $MnCl_2$, 2 mM dithiothreitol, 5 mM AcP, 0.01 mM sodium orthovanadate). After 20 min at 37°C (or 1h at room temperature for $PorX_{FJ}$ D54A/T271V due to precipitation issues), the reactions were quenched by flash-freezing in liquid nitrogen.

Protein dimerization assay *in vitro*

$PorX_{FJ}$ variants (100 μM wildtype or mutants) were incubated in buffer E supplemented with either 300 μM $ZnCl_2$ or 20 mM acetyl phosphate (AcP) and 5 mM $MgCl_2$ for 1 h at room temperature. The mixture was then subjected to size exclusion chromatography (Superdex 200 increase 10/300 GL or Superdex 75 increase 10/300 GL), pre-equilibrated with buffer E.

Functional characterization in *F. johnsoniae*

Bacterial strains, plasmids, and growth conditions

F. johnsoniae UW101 (4, 5) was the wild-type strain used in this study. *F. johnsoniae* strains were grown at 25° in CYE liquid (11) or TYES (12). For solid media, 15 g agar was added per liter. *E. coli* strains were grown in Luria-Bertani medium (LB) at 37°C (13). For most experiments, *F. johnsoniae* strains were propagated from −80 ^{o}C glycerol stocks on CYE agar and incubated for 48-72 h at 25°C before they were used as starter cultures. All primers and plasmids used in this study are listed in Table S1 and S2, respectively. Antibiotics were used at the following concentrations when needed: ampicillin, 100 μg/ml; erythromycin, 100 μg/ml; kanamycin, 35 μg/ml; and tetracycline, 20 μg/ml unless indicated otherwise.

Construction of the deletion mutants in *F. johnsoniae*

For deletion of $porX_{FJ}$ (Fjoh_2906), a 2.0-kbp fragment spanning part of Fjoh_2905 and the first 105 bp of $porX_{FJ}$ was amplified using primers 0076 (introducing a BamHI site) and 0077 (introducing an XbaI site), and the Phusion DNA polymerase (Thermo Fisher Scientific, Waltham,

MA). The fragment was digested with BamHI and XbaI and ligated into pYT313 (14), which had been digested with the same enzymes, to generate pIM03. A 1.9-kbp fragment spanning Fjoh_2907, Fjoh_2908, and the last 45 bp of *porX*$_{FJ}$ was amplified using primers 0167 (introducing an XbaI site) and 0079 (introducing a SalI site). The fragment was cloned into XbaI and SalI sites of pIM03 to generate the deletion construct pIM06. Plasmid pIM06 was introduced into *F. johnsoniae* UW101 by triparental conjugation as previously described (15). An erythromycin-resistant clone was streaked for isolation, and grown overnight in CYE liquid with shaking at 25°C in the absence of antibiotics. These cells were plated on CYE agar containing 5% sucrose and incubated at 25°C for 2-3 days. Sucrose-resistant colonies were streaked for isolation and screened by PCR using primers 0086 and 0087, which flank *porX*$_{FJ}$, to identify the deletion mutant CJ4057. The same procedure was used to delete *gldKLMNO* using the plasmids and primers listed in Table S1 and Table S2, respectively.

Complementation of the *porX* deletion mutant

Primers 0086 (introducing a BamHI site) and 0087 (introducing an XbaI site) were used to amplify a 2009-bp fragment spanning *porX* with its putative promoter. The fragment was digested with BamHI and XbaI and ligated into pCP23 (8), which had been digested with the same enzymes, to generate pIM10. The plasmid was transferred to the *porX* mutant by triparental conjugation. Tetracycline was used for screening of the complemented colonies.

Analysis of cell motility

Cells of the wild type *F. johnsoniae* and *porX*$_{FJ}$ deletion mutant were grown for 17 h at 25°C in motility medium (16) without shaking. Tunnel slides were constructed using double stick tape, glass microscope slides, and glass coverslips, as previously described (17). Ten microliters of cultures were introduced into the tunnel slides, incubated for 5 min, and observed for motility using an Olympus CX41 phase-contrast microscope at room temperature (22°C). Images were recorded using a Moment CMOS camera and analyzed using Ocular (Teledyne Photometrics, Tuscon, AZ). Rainbow traces of cell movements were made using Fiji (https://imagej.net/) and the macro–Color FootPrint (18).

Growth of *F. johnsoniae* on chitin

Chitin powder (practical grade from shrimp shells; Sigma C7170) was prepared as a 1% slurry essentially as described previously (19) and used as a stock solution to prepare the chitin media. Cells of *F. johnsoniae* were streaked on fresh TYES agar and incubated at 25°C for 2 d. Cells were scraped off the plates, suspended in 1 ml Stanier medium (20), pelleted by centrifugation at 4,200 × g for 3 min to remove the residual TYES medium, and resuspended in Stanier medium to a concentration (OD_{600}) of 1.0. Then 0.1 ml of the inoculation cell suspension was introduced into 50 ml of Stanier medium supplemented with 0.05% (w/v) chitin in 250-ml flasks, and incubated at 25°C with shaking (200 rpm). 2.5 µg/ml of tetracycline was added for growth of the complemented strain. At various times, 1 ml samples were removed. Cells and residual chitin were collected by centrifugation at 17,000 × g for 10 min. Growth was assessed by determining the total cellular protein in the pellets using the Bradford assay as previously described (21).

References:

1. C. Matsumoto-Mashimo, A. M. Guerout, D. Mazel, A new family of conditional replicating plasmids and their cognate Escherichia coli host strains. *Res Microbiol* **155** (2004).

2. F. Bolivar, K. Backman, Plasmids of Escherichia coli as cloning vectors. *Methods Enzymol* **68** (1979).

3. D. H. Figurski, D. R. Helinski, Replication of an origin-containing derivative of plasmid RK2 dependent on a plasmid function provided in trans. *Proc Natl Acad Sci U S A* **76** (1979).

4. M. J. McBride, *et al.*, Novel features of the polysaccharide-digesting gliding bacterium Flavobacterium johnsoniae as revealed by genome sequence analysis. *Appl Environ Microbiol* **75** (2009).

5. M. J. McBride, T. F. Braun, GldI Is a Lipoprotein that Is Required for Flavobacterium johnsoniae Gliding Motility and Chitin Utilization. *J Bacteriol* **186** (2004).

6. A. M. Lasica, *et al.*, Structural and functional probing of PorZ, an essential bacterial surface component of the type-IX secretion system of human oral-microbiomic Porphyromonas gingivalis. *Sci Rep* **6**, 1–22 (2016).

7. Y. Zhu, *et al.*, Genetic analyses unravel the crucial role of a horizontally acquired alginate lyase for brown algal biomass degradation by Zobellia galactanivorans. *Environ Microbiol* **19**, 2164–2181 (2017).

8. S. Agarwal, D. W. Hunnicutt, M. J. Mcbride, Cloning and characterization of the Flavobacterium johnsoniae (Cytophaga johnsonae) gliding motility gene, gldA. *Proc Natl Acad Sci U S A* **94**, 12139–12144 (1997).

9. F. Madeira, *et al.*, Search and sequence analysis tools services from EMBL-EBI in 2022. *Nucleic Acids Res* **50**, gkac240–gkac240 (2022).

10. X. Robert, P. Gouet, Deciphering key features in protein structures with the new ENDscript server. *Nucleic Acids Res* **42**, W320–W324 (2014).

11. M. J. Mcbride, M. J. Kempf, Development of techniques for the genetic manipulation of the gliding bacterium Cytophaga johnsonae. *J Bacteriol* **178**, 583–590 (1996).

12. K. D. Cain, B. R. LaFrentz, Laboratory Maintenance of Flavobacterium psychrophilum and Flavobacterium columnare. *Curr Protoc Microbiol* **6** (2007).

13. J. Sambrook, E. F. Fritsch, T. Maniatis, *In: Molecular Cloning: A Laboratory Manual, Cold Spring Harbor Laboratory, Cold Spring Harbor, New York.* (1989).

14. Y. Zhu, *et al.*, Genetic analyses unravel the crucial role of a horizontally acquired alginate lyase for brown algal biomass degradation by Zobellia galactanivorans. *Environ Microbiol* **19** (2017).

15. D. W. Hunnicutt, M. J. McBride, Cloning and characterization of the Flavobacterium johnsoniae gliding motility genes gldD and gldE. *J Bacteriol* **183** (2001).

16. J. Liu, M. J. McBride, S. Subramaniam, Cell surface filaments of the gliding bacterium Flavobacterium johnsoniae revealed by cryo-electron tomography. *J Bacteriol* **189** (2007).

17. A. Shrivastava, R. G. Rhodes, S. Pochiraju, D. Nakane, M. J. McBride, Flavobacterium johnsoniae RemA is a mobile cell surface lectin involved in gliding. *J Bacteriol* **194** (2012).

18. D. Nakane, K. Sato, H. Wada, M. J. McBride, K. Nakayama, Helical flow of surface protein required for bacterial gliding motility. *Proc Natl Acad Sci U S A* (2013). https://doi.org/10.1073/pnas.1219753110.

19. M. Dworkin, S. Falkow, E. Rosenberg, K.-H. Schleifer, E. Stackebrandt, *The Prokaryotes A Handbook on the Biology of Bacteria (Third Edition Volume 5: Proteobacteria: Alpha and Beta Subclasses)* (2006).

20. R. Y. Stanier, The Cytophaga Group: A Contribution To The Biology Of Myxobacteria. *Bacteriol Rev* **6** (1942).

21. Y. Zhu, *et al.*, Outer membrane proteins related to SusC and SusD are not required for Cytophaga hutchinsonii cellulose utilization. *Appl Microbiol Biotechnol* **99** (2015).
